# Subacute Oral Toxicity Study of a New Type of Cordyceps, *Paecilomyces sinclairii*, in Sprague-Dawley Rats

**DOI:** 10.5487/TR.2009.25.2.101

**Published:** 2009-06-01

**Authors:** Seung Jun Kwack, Byung Mu Lee

**Affiliations:** 16 Department of Toxicological Research, National Institute of Toxicological Research, Seoul, 122-704 Korea; 26 Division of Toxicology, College of Pharmacy, Sungkyunkwan University, Chunchun-dong 300, Changan-ku, Suwon, Gyeonggi-do, 440-746 Korea

**Keywords:** 2 week-oral toxicity, *Paecilomyces sinclairii*, Sprague-Dawley rats

## Abstract

This study was conducted to investigate the 2 week-oral toxicity of *Paecilomyces sinclairii* in Sprague-Dawley rats. *P. sinclairii* was daily administered to male and female rats for 2 weeks with different dose levels (0, 0.008, 0.04, 0.2, 1 and 5 g/kg). There were no clinical signs compared with control group, but a slight increase of white blood cell (WBC) was observed in the males rats receiving all dose levels of *P. sinclairii*. In biohematological analysis, the levels of glucose and cholesterol in the blood were decreased slightly in the males and females rats at doses of 0.008 or 1 g/kg. At the all dose groups, there were no significant changes in the body weights, but autopsy findings of all organs showed reduced weights in the thymus of males in the high dose groups of 1 g/kg and 5 g/kg. These results indicate that *P. sinclairii* does not induce any significant toxic effect on Sprague-Dawley rats treated for 2 weeks, but the reduced weights of thymus in males may require a further long-term investigation.

## INTRODUCTION

Cordyceps, particularly *Cordyceps sinensis*, an entomogenous fungus on the larvae and pupae of insects, is highly valued as a health food due to its various biological functions, and has been regarded as a potential drug in China and is currently used as a tonic (Furuya *et al*., 1983; Shin *et al*., 2003). *C. sinensis* and the cultured mycelia of five species of Cordyceps and four species of Isaria were found to inhibit the twitch response of guinea pig ileum and the aggregation of human blood platelets. It is suggested that these activities are due to a combination of adenosine, 5'-adenosine monophophate and several other nucleic acid-related compounds, present in the extracts (Ikumoto *et al*., 1991).

Pharmacological function of Cordyceps has been known as anti-tumour, immunostimulation, hypoglycemic, lowering of cholesterol and inhibition of peroxisome proliferation (Furuya *et al*., 1983; Liu *et al*., 1991; Kiho *et al*., 1993, 1996; Kuo *et al*., 1994). A type of *Cordyceps* sp., *Paecilomyces japonica* fungus, cultivated from a fungus-infected silk worm larvae, showed anti-tumourigenic and immunostimulating activities by elevating free radical scavenging enzymes and/or by inhibiting lipid peroxidation (Shin *et al*., 2003).

*Isaria sinclairii* has been recently introduced in powdered form as a new potential Korean crude drug. It contains the fruiting bodies of *I. sinclairii* and its parasitic host larva. *I. sinclairii* showed immunosuppressive activity by inducing apoptosis in lymphocytes (Fujino *et al*., 2001, 2002). Moreover, the fruiting bodies of Isaria fungi have been used to treat cancer patients in Korea (Oh *et al*., 2001). Drink products containing of *I. sinclairii* or *P. jaonica* are already commercially available in Korea and *I. sinclairii* is considered as a candidate nutraceutical for diabetics and to be a chemopreventive agent.

*Paecilomyces sinclairii*, a new type of Cordyceps sp., has been isolated from cicada larvae and mass production of this strain through artificial cultivation have recently been established (Kim *et al.*, 2003; Shin *et al.*, 2003). Therefore, it is necessary to study the toxicity of fruit bodies of silk-worm larvae infected with *P. sinclairii*. The present study was aimed to study the oral toxicity of the powder of *P. sinclairii* in rats performed as a part of a safety evaluation of a new type of Cordyceps as a functional food material.

## MATERIALS AND METHODS

***Experimental animals and housing conditions.*** Young adult SD rats (4 week-old) of both sexes were purchased from the Jeil Laboratory Animals (Seoul, Korea) and acclimated for 1 week prior to commencement of the test. They were housed in rectangular polycarbonate cages according to group and sex under controlled conditions of temperature (23 ± 3°C), humidity (55 ± 5%), ventilation (10~18 times per one hour), light (12 : 12 h, light : dark cycle) and illumination (300~500 Lux). They were fed with pellets along with free access to tap water. The animals were randomly allocated to one control group and five treatment groups, each consisting of five males and five females (Landsdown, 1993; Rehg and Toth, 1998).

***Treatments.*** *P. sinclairii* was obtained from the National Institute of Agricultural Science and Technology (Suwon, Korea) and prepared as powder. The test powder was suspended in double distilled water and was prepared daily immediately before the treatment and directly administered to the stomach of the animals using a gastric tube in a dose of 0.008, 0.04, 0.2, 1 and 5 g/kg body weight (bw)/day, respectively; 1 ml/100 g bw of double distilled water alone was fed orally to the animals in the control group. The treated dose was determined by the previous acute oral toxicity study (Ahn *et al.*, 2003).

***Clinical signs and mortality.*** The animals were observed thoroughly for the onset of any immediate toxic signs, also during the observation period to record any delayed acute effects and mortality. The animals were observed at 10 min, 30 min, 1 h, 2 h, 4 h, 6 h and 1 times per one day for 14 days after administration of the powder of *P. sinclairii* (Lorke, 1983).

***Body weights and dietary consumption.*** The animals were monitored for body weight changes 0, 3, 6, 9, 12 and 14 days after administration of the tested powder. The mean of body weight was calculated in the animals that survived up to the end of the period (14 days). The dietary consumption for each group was measured at the start of treatment and during the 2 weeks of treatment period (Kim *et al.*, 1993; Schlede *et al.*, 1995).

***Hematology and serum biochemistry.*** During the autopsy, whole blood taken from all rats from each group was allowed to clot and the serum was separated and the blood samples for hematological analysis were collected into CBC bottles containing EDTA-2K (Green Cross Medical Industry, Korea). Red blood cell count (RBC), hemoglobin concentration, hematocrit, mean corpuscular volume (MCV), mean corpuscular hemoglobin (MCH), mean corpuscular hemoglobin concentration (MCHC), platelet count and white blood cell count (WBC) were determined using a Coulter counter T-540 (Coulter Counter Electronics, USA). For serum biochemistry analysis, blood samples were centrifuged at 3000 rpm for 10 min within 1h after collection. The sera were stored in the -80°C freezer before they were analyzed. Serum biochemistry parameters including calcium, potassium, sodium, albumin, blood urea nitrogen (BUN), cholesterol, creatinine, glucose, total cholesterol, total bilirubin, total protein, alkaline phosphatase (ALP), glutamate pyruvate (GPT), glutamate oxaloacetate transaminase (GOT), γ-gluamyl transferase (GGT) and creatine phosphokinase (CPK) were evaluated by an autoanalyzer, model Hitachi 7170 (Tokyo, Japan) (Mellert *et al.*, 2003; Kitamura *et al.*, 2003; Kanki *et al.*, 2003).

***Organ weights.*** Under anesthesia, the following vital organs including kidney, heart, liver, lung, spleen and thymus were isolated and measured. (Kim *et al.*, 1996 ; Kwack *et al.*, 1996; Long *et al.*, 1998).

***Autopsy and observed opinion.*** All animals were killed under anesthesia after 2 weeks and following organs were observed: kidney, heart, liver, lung, spleen, adrenal glands, thyroid glands, testes, prostates, ovaries and uterus.

***Determination of $LD_{50}$.*** The number of rats that died within the period of study was noted for each group and subsequently the $LD_{50}$ was calculated by Litchfield and Wilcoxon analysis (Litchfield and Wilcoxon, 1949).

***Statistical analysis.*** Quantitative differences between control group and treat group in body weights, blood biochemistry and organ weights were statistically analyzed using Student's *t*-test. *P* values of <0.05 were considered statistically significant.

## RESULTS

***Clinical signs and mortality.*** There was no treatment-related mortality in animals treated with *P. sinclairii* at dose of 0, 0.008, 0.04, 0.2, 1 and 5 g/kg bw/day for 2 weeks (data not shown).

***Body weights changes.*** The body weights of both sexes that were fed with *P. sinclairii* at various concentrations (0.008, 0.04, 0.2, 1 and 5 g/kg bw/day) were slightly increased but were statistically not significant when compared with the control group during 2 weeks. Considering the pattern of weight gain in both sexes, the total average weight gain in females was lower compared with males (Fig. 1 and 2).

***Food consumption.*** In males, food consumption was slightly decreased on day 8 and 9 in the 0.04, 0.2 and 1 g/kg bw/day treatments when compared with the control group, but recovered on day 12 after administration (Fig. 3 and 4).

***Hematology.*** As shown in Table 1, there were no statistically significant differences in RBC, hemoglobin, Ht, MCV, MHC, MCHC and platelet counts of both sexes between the control and treatment groups. Total WBC of males were decreased in the all treated groups when compared with the control group, but there were no statistically significant differences and all values were in the normal range.

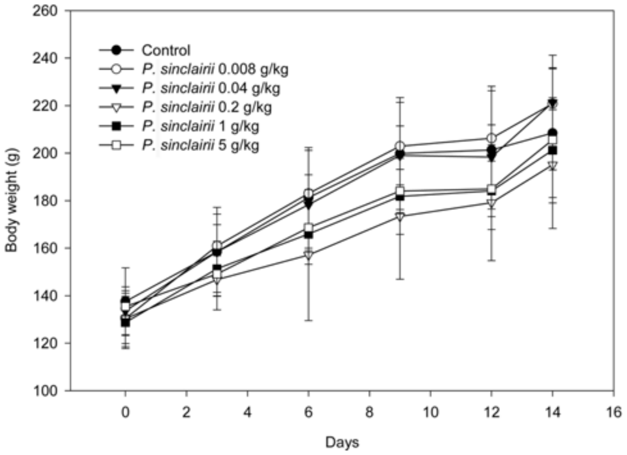

**Fig. 1.** Body weight changes in Sprague-Dawley male rats orally administered with *P. sinclairii* for 2 weeks.

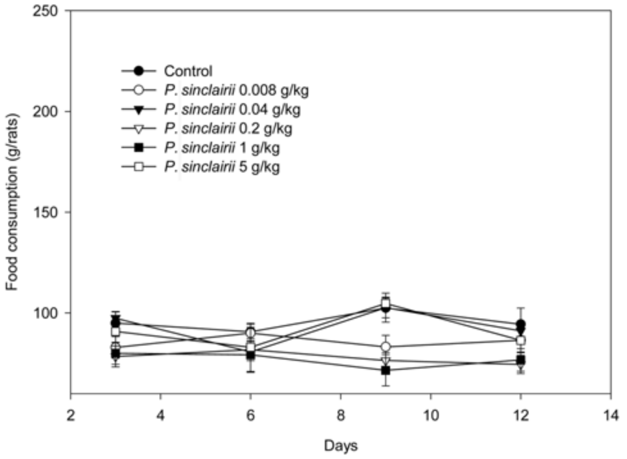

**Fig. 2.** Body weight changes in Sprague-Dawley female rats orally administered with *P. sinclairii* for 2 weeks.

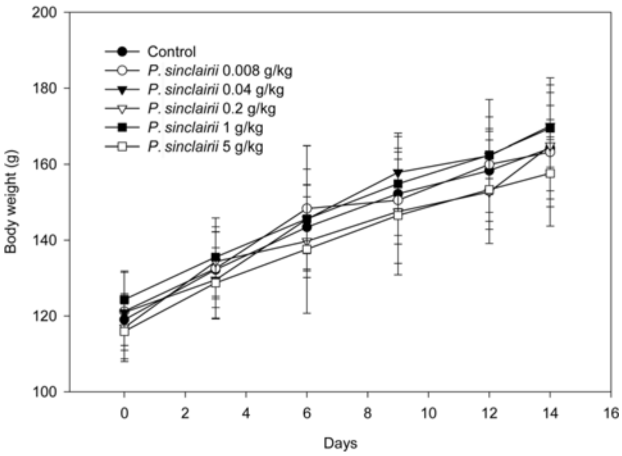

**Fig. 3.** Food consumption of Sprague-Dawley male rats orally administered with *P. sinclairii* for 2 weeks.

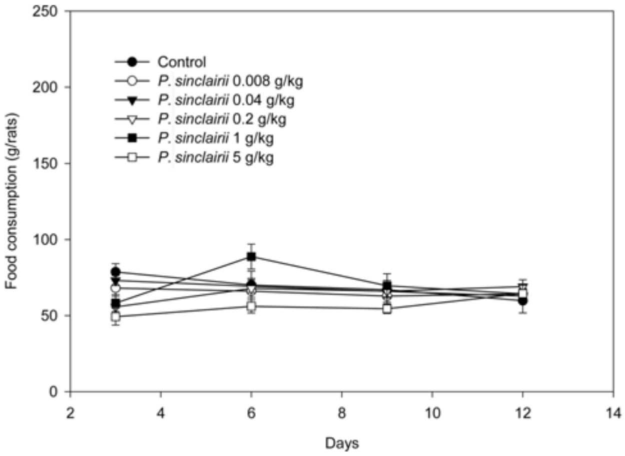

**Fig. 4.** Food consumption of Sprague-Dawley female rats orally administered with *P. sinclairii* for 2 weeks.

**Table 1.** Hematological values of Sprague-Dawley rats orally treated with *Paecilomyces sinclairii*[a]

| Parameter | CON[b] | Group 1 (0.008 g/kg) | Group 2 (0.04 g/kg) | Group 3 (0.2 g/kg) | Group 4 (1 g/kg) | Group 5 (5 g/kg) |
|---|---|---|---|---|---|---|
| Males | | | | | | |
| WBC ($10^3$/mm$^3$) | 10.8 ± 0.2 | 11.1 ± 0.4 | 11.6 ± 0.3 | 11.0 ± 0.7 | 12.1 ± 0.8 | 11.0 ± 0.3 |
| RBC ($10^6$/mm$^3$) | 6.5 ± 0.2 | 6.2 ± 0.7 | 6.5 ± 0.5 | 6.3 ± 0.3 | 5.9 ± 0.7 | 6.0 ± 0.9 |
| Hb (g/dl) | 12.8 ± 0.8 | 11.9 ± 1.5 | 13.0 ± 0.9 | 11.7 ± 1.3 | 12.3 ± 0.6 | 12.0 ± 1.7 |
| Ht (%) | 49.7 ± 5.3 | 47.7 ± 3.2 | 48.8 ± 3.3 | 48.1 ± 4.9 | 47.4 ± 1.4 | 45.9 ± 2.9 |
| MCV ($\mu^3$) | 76.5 ± 9.4 | 77.5 ± 7.9 | 75.7 ± 7.4 | 80.1 ± 6.1 | 82.6 ± 4.7 | 78.1 ± 6.1 |
| MCH (pg) | 20.3 ± 1.6 | 19.2 ± 1.0 | 20.2 ± 1.7 | 19.1 ± 1.4 | 21.9 ± 2.0 | 20.1 ± 1.8 |
| MCHC (g/dl) | 26.7 ± 1.5 | 25.1 ± 3.4 | 26.7 ± 1.7 | 24.3 ± 2.5 | 25.8 ± 1.6 | 26.1 ± 3.2 |
| PLT ($10^3$/mm$^3$) | 818.6 ± 15.2 | 812.2 ± 59.1 | 819.4 ± 66.6 | 798.0 ± 45.6 | 837.0 ± 18.8 | 833.8 ± 91.0 |
| Females | | | | | | |
| WBC ($10^3$/mm$^3$) | 12.8 ± 1.0 | 12.1 ± 1.0 | 12.9 ± 1.2 | 12.2 ± 0.8 | 12.7 ± 2.7 | 12.3 ± 1.8 |
| RBC ($10^6$/mm$^3$) | 5.3 ± 0.8 | 6.6 ± 0.2 | 6.2 ± 0.5 | 5.6 ± 0.4 | 6.5 ± 0.5 | 5.2 ± 1.1 |
| Hb (g/dl) | 10.5 ± 0.7 | 13.9 ± 1.1 | 12.3 ± 0.7 | 11.8 ± 1.1 | 13.1 ± 0.6 | 10.8 ± 1.3 |
| Ht (%) | 44.3 ± 2.8 | 45.8 ± 0.5 | 43.4 ± 2.2 | 41.4 ± 4.2 | 44.6 ± 3.8 | 38.2 ± 6.6 |
| MCV ($\mu^3$) | 88.5 ± 11.0 | 69.1 ± 1.9 | 71.7 ± 7.3 | 73.4 ± 7.3 | 68.9 ± 5.2 | 73.9 ± 14.1 |
| MCH (pg) | 21.6 ± 2.0 | 20.5 ± 0.2 | 19.9 ± 1.0 | 20.9 ± 0.5 | 20.2 ± 0.8 | 20.0 ± 0.7 |
| MCHC (g/dl) | 24.4 ± 2.1 | 29.7 ± 1.1 | 27.1 ± 2.4 | 28.8 ± 2.9 | 29.4 ± 1.9 | 27.6 ± 4.4 |
| PLT ($10^3$/mm$^3$) | 872.8 ± 99.1 | 805.2 ± 45.8 | 848.4 ± 45.7 | 827.6 ± 83.4 | 892.4 ± 60.2 | 816.2 ± 54.3 |

[a]Mean ± S.D.
[b]Treated with distilled water.

**Table 2.** Biochemical serum values of male Sprague-Dawley rats orally treated with *Paecilomyces sinclairii*[a]

| Parameters | CON[b] | Group 1 (0.008 g/kg) | Group 2 (0.04 g/kg) | Group 3 (0.2 g/kg) | Group 4 (1 g/kg) | Group 5 (5 g/kg) |
|---|---|---|---|---|---|---|
| TP (g/dl) | 6.0 ± 0.5 | 6.2 ± 0.1 | 6.0 ± 0.3 | 6.5 ± 0.2 | 6.2 ± 0.4 | 6.0 ± 0.2 |
| ALB (g/dl) | 2.3 ± 0.2 | 2.4 ± 0.1 | 2.3 ± 0.1 | 2.6 ± 0.4 | 2.4 ± 0.2 | 2.4 ± 0.0 |
| GLU (g/dl) | 105.0 ± 15.3 | 101.0 ± 11.3 | 101.4 ± 7.1 | 100.6 ± 6.6 | 103.4 ± 5.1 | 104.4 ± 10.7 |
| T.Chol (mg/dl) | 69.6 ± 9.6 | 59.4 ± 10.8 | 65.4 ± 8.6 | 71.0 ± 5.6 | 64.8 ± 4.4 | 63.0 ± 3.7 |
| T.Bil (mg/dl) | 0.2 ± 0.1 | 0.3 ± 0.1 | 0.3 ± 0.2 | 0.4 ± 0.3 | 0.3 ± 0.2 | 0.4 ± 0.1 |
| GGT (IU/l) | 1.4 ± 0.5 | 1.6 ± 0.9 | 1.8 ± 0.8 | 1.6 ± 0.5 | 1.4 ± 0.5 | 1.4 ± 0.5 |
| GPT (IU/l) | 40.4 ± 3.0 | 42.0 ± 4.3 | 43.0 ± 4.3 | 47.0 ± 3.7 | 47.6 ± 3.8 | 50.2 ± 4.1 |
| GOT (IU/l) | 118.0 ± 12.5 | 106.8 ± 19.1 | 106.0 ± 8.9 | 101.2 ± 9.3 | 100.8 ± 4.3 | 102.2 ± 5.1 |
| ALP (IU/l) | 155.2 ± 16.2 | 153.4 ± 13.7 | 151.8 ± 15.0 | 159.2 ± 17.3 | 159.2 ± 11.5 | 155.6 ± 16.9 |
| BUN (mg/l) | 17.5 ± 1.5 | 17.5 ± 1.8 | 17.5 ± 1.1 | 17.7 ± 1.8 | 15.9 ± 0.9 | 15.6 ± 1.9 |
| CRN (mg/dl) | 0.6 ± 0.1 | 0.6 ± 0.1 | 0.6 ± 0.1 | 0.5 ± 0.1 | 0.6 ± 0.1 | 0.5 ± 0.0 |
| CPK (IU/l) | 708.2 ± 89.8 | 662.6 ± 41.9 | 668.0 ± 73.9 | 799.8 ± 38.2 | 685.4 ± 65.9 | 672.4 ± 36.5 |
| Na (mEQ/l) | 145.3 ± 1.6 | 142.0 ± 4.8 | 144.4 ± 1.7 | 144.9 ± 2.3 | 143.1 ± 1.9 | 142.5 ± 2.3 |
| K (mEQ/l) | 5.5 ± 1.0 | 7.7 ± 0.7 | 5.3 ± 0.8 | 7.5 ± 2.1 | 6.7 ± 1.0 | 6.5 ± 1.0 |
| Cl (mEQ/l) | 102.5 ± 1.3 | 101.5 ± 2.5 | 100.4 ± 1.5 | 104.3 ± 3.6 | 101.4 ± 1.3 | 102.5 ± 0.9 |
| Ca (mg/l) | 10.6 ± 0.5 | 10.7 ± 0.9 | 10.3 ± 0.8 | 10.7 ± 0.6 | 10.8 ± 0.8 | 11.5 ± 0.8 |

[a]Mean ± S.D.
[b]Treated with distilled water.

***Serum biochemistry.*** In both sexes, blood glucose and total cholesterol levels were slightly decreased in the 0.008 g/kg bw/day treatment, and blood glucose level was decreased in the female 1 g/kg bw/day group when compared with the control group, but the values were within the normal range. Also, GGT, GPT, GOT, total protein, albumin, BUN, CPK, creatinine, calcium, potassium and sodium values were no significant differences between the control and treatment groups (Table 2 and 3).

***Organ weights, Autopsy and observed opinion.*** The results for absolute organ weights are shown in Table 4. No treatment-dependent variation was observed. At autopsy, observation of the organs did not show any change due to the treatment of *P. sinclairii.*

## DISCUSSION

The present study was conducted to investigate the potential repeated oral toxicity of *P. sinclairii* adminis-

**Table 3.** Biochemical serum values of female Sprague-Dawley rats orally treated with *Paecilomyces sinclairii*[a]

| Parameters | CON[b] | Group 1 (0.008 g/kg) | Group 2 (0.04 g/kg) | Group 3 (0.2 g/kg) | Group 4 (1 g/kg) | Group 5 (5 g/kg) |
|---|---|---|---|---|---|---|
| TP (g/dl) | 6.5 ± 0.5 | 7.0 ± 0.9 | 6.6 ± 0.4 | 6.6 ± 0.4 | 6.1 ± 0.2 | 7.1 ± 0.2 |
| ALB (g/dl) | 2.5 ± 0.3 | 3.1 ± 1.0 | 2.5 ± 0.2 | 2.4 ± 0.3 | 2.4 ± 0.2 | 2.5 ± 0.2 |
| GLU (g/dl) | 117.2 ± 11.3 | 115.6 ± 12.4 | 118.2 ± 18.9 | 119.2 ± 13.8 | 101.0 ± 11.1 | 111.8 ± 19.0 |
| T.Chol (mg/dl) | 71.8 ± 6.1 | 65.8 ± 5.9 | 68.2 ± 6.3 | 73.0 ± 7.3 | 70.0 ± 14.1 | 63.0 ± 4.6 |
| T.Bil (mg/dl) | 0.4 ± 0.2 | 0.5 ± 0.1 | 0.3 ± 0.1 | 0.3 ± 0.2 | 0.3 ± 0.1 | 0.3 ± 0.1 |
| GGT (IU/l) | 1.8 ± 0.4 | 1.6 ± 0.5 | 1.6 ± 0.5 | 1.2 ± 0.4 | 1.4 ± 0.5 | 1.4 ± 0.5 |
| GPT (IU/l) | 41.4 ± 4.9 | 43.0 ± 4.9 | 41.4 ± 5.3 | 41.8 ± 4.2 | 37.4 ± 3.6 | 41.2 ± 4.8 |
| GOT (IU/l) | 115.0 ± 6.8 | 120.0 ± 7.5 | 121.8 ± 14.4 | 120.0 ± 9.0 | 120.6 ± 5.2 | 116.4 ± 7.8 |
| ALP (IU/l) | 108.6 ± 14.0 | 108.0 ± 10.2 | 109.2 ± 5.6 | 112.2 ± 8.5 | 116.4 ± 9.3 | 107.0 ± 12.7 |
| BUN (mg/l) | 17.5 ± 2.0 | 18.9 ± 3.7 | 16.2 ± 3.9 | 16.0 ± 4.1 | 15.4 ± 2.6 | 17.3 ± 0.8 |
| CRN (mg/dl) | 0.6 ± 0.1 | 0.6 ± 0.2 | 0.6 ± 0.1 | 0.6 ± 0.0 | 0.5 ± 0.1 | 0.7 ± 0.1 |
| CPK (IU/l) | 685.8 ± 41.9 | 704.4 ± 74.4 | 701.2 ± 87.1 | 725.0 ± 88.9 | 796.2 ± 92.1 | 752.8 ± 81.1 |
| Na (mEQ/l) | 137.9 ± 1.2 | 139.1 ± 2.5 | 139.0 ± 2.6 | 140.4 ± 2.0 | 136.4 ± 1.2 | 136.3 ± 1.4 |
| K (mEQ/l) | 6.6 ± 1.3 | 5.1 ± 0.7 | 5.3 ± 1.0 | 5.3 ± 1.0 | 5.8 ± 0.8 | 7.6 ± 2.1 |
| Cl (mEQ/l) | 102.0 ± 1.6 | 100.7 ± 0.5 | 101.6 ± 0.9 | 102.3 ± 0.4 | 100.0 ± 1.9 | 100.6 ± 0.4 |
| Ca (mg/l) | 10.6 ± 0.7 | 9.8 ± 0.8 | 10.6 ± 0.5 | 10.7 ± 0.2 | 10.0 ± 0.5 | 9.7 ± 1.5 |

[a]Mean ± S.D.
[b]Treated with distilled water.

**Table 4.** Absolute organ weights of Sprague-Dawley rats orally treated with *Paecilomyces sinclairii*[a]

| Parameters | | Lung | Heart | Kedney | Liver | Spleen | Thymus |
|---|---|---|---|---|---|---|---|
| Male | CON[b] | 1.34 ± 0.02 | 0.83 ± 0.03 | 1.63 ± 0.12 | 9.62 ± 0.84 | 1.40 ± 0.18 | 0.53 ± 0.04 |
| | Group 1 (0.008 g/kg) | 1.37 ± 0.10 | 0.86 ± 0.08 | 1.59 ± 0.20 | 9.29 ± 0.57 | 1.46 ± 0.15 | 0.46 ± 0.05 |
| | Group 2 (0.04 g/kg) | 1.29 ± 0.10 | 0.82 ± 0.08 | 1.58 ± 0.12 | 9.75 ± 0.52 | 1.46 ± 0.09 | 0.44 ± 0.06 |
| | Group 3 (0.2 g/kg) | 0.97 ± 0.18* | 0.73 ± 0.07 | 1.41 ± 0.14 | 8.91 ± 0.73 | 1.47 ± 0.19 | 0.44 ± 0.03 |
| | Group 4 (1 g/kg) | 1.03 ± 0.13 | 0.75 ± 0.05 | 1.55 ± 0.14 | 9.18 ± 0.90 | 1.38 ± 0.13 | 0.39 ± 0.01* |
| | Group 5 (5 g/kg) | 1.21 ± 0.19 | 0.73 ± 0.07 | 1.45 ± 0.16 | 9.26 ± 0.56 | 1.31 ± 0.14 | 0.35 ± 0.04* |
| Female | CON | 1.01 ± 0.05 | 0.61 ± 0.01 | 1.12 ± 0.07 | 6.77 ± 0.37 | 1.14 ± 0.12 | 0.34 ± 0.03 |
| | Group 1 (0.008 g/kg) | 1.23 ± 0.16 | 0.60 ± 0.06 | 1.25 ± 0.19 | 6.31 ± 0.74 | 1.09 ± 0.15 | 0.35 ± 0.03 |
| | Group 2 (0.04 g/kg) | 1.07 ± 0.18 | 0.65 ± 0.06 | 1.19 ± 0.17 | 6.72 ± 0.83 | 1.25 ± 0.15 | 0.33 ± 0.08 |
| | Group 3 (0.2 g/kg) | 1.26 ± 0.11 | 0.65 ± 0.07 | 1.17 ± 0.14 | 6.81 ± 0.79 | 1.11 ± 0.13 | 0.37 ± 0.05 |
| | Group 4 (1 g/kg) | 1.16 ± 0.13 | 0.61 ± 0.06 | 1.23 ± 0.11 | 6.67 ± 0.19 | 0.90 ± 0.12 | 0.39 ± 0.05 |
| | Group 5 (5 g/kg) | 1.10 ± 0.12 | 0.56 ± 0.04 | 1.11 ± 0.06 | 6.00 ± 0.69 | 1.00 ± 0.09 | 0.35 ± 0.06 |

[a]Mean ± S.D.
[b]Treated with distilled water.

tered by gavage to Sprague-Dawley rats at dose levels of 0, 0.008, 0.04, 02, 1 and 5 g/kg bw/day for 2 weeks. 2 week after administration of *P. sinclairii,* the clinical signs, mortalities, body weight changes, hematology, blood biochemistry and absolute organ weights were examined.

There were no mortalities and clinical signs of toxicity either immediately or during the post-treatment period. In hematological analysis, total WBC of males were decreased in the all treated groups when compared with the control group, but there were no statistically significant differences and all values were in the normal range. In serum analysis, blood glucose and total cholesterol values were slightly decreased in some treated group when compared with the control group, but there were also in the normal range.

The statistically significant changes of biochemical parameters in the serum of rats were not showed when compared with control group and these results suggested that *P. sinclairii* causes no disruption of normal physiological and biochemical homeostasis. However, administration of *P. sinclairii* reduced the absolute organ weights of thymus in males treated with 1 g/kg and 5 g/kg bw/day.

In conclusion, the results of the present study showed that 2 weeks repeated oral toxicity of *P. sinclairii* was not significant in the rats of both sexes and $LD_{50}$ value was greater than 5 g/kg bw/day in to Sprague-Dawley rats. However, the reduced weights of thymus in males may require a further long-term toxicological evaluation.

## Abbreviations

RBC: red blood cell count
Hb: hemoglobin concentration
Ht: hematocrit
MCV: mean corpuscular volume
MCHC: mean corpuscular hemoglobin concentration
PLT: platelets
WBC: white blood cell count
TP: total protein
ALB: albumin
GLU: glucose
T.Chol: total cholesterol
T.Bil: total bilirubin
GGT: γ-glutamyl transferase
GPT: glutamate pyruvate transaminase
GOT: glutamate oxaloacetate transaminase
ALP: alkaline phosphatase
BUN: blood urea nitrogen
CRN: creatinine
CPK: creatine phophokinase
Na: sodium
K: potassium
CI: chloride
Ca: calcium
